# IFITM1-targeted NIR-II fluorescence imaging enables visualisation of colorectal cancer and metastatic lymph nodes

**DOI:** 10.1186/s12967-026-07938-0

**Published:** 2026-03-24

**Authors:** Shangkun Jin, Ruirong Lin, Shen Guan, Jiantao Jiang, Hongxin He, Enhao Wei, Ziyuan Lv, Weijin Yang, Jingtao Huang, Shiyao Zheng, Zhihua Zhou, Ruru Ge, Xiaohua Jia, Chunkang Yang

**Affiliations:** 1 Clinical Oncology School of Fujian Medical University, Fujian Cancer Hospital, Fuzhou, Fujian 350014 P.R. China; 2 Department of Colorectal Surgery, Clinical Oncology School of Fujian Medical University, Fujian Cancer Hospital, Fuzhou, Fujian 350014 P.R. China; 3 College of Chemistry, Fuzhou University, Fuzhou, Fujian 350116 China; 4 Department of Clinical Medicine, Shanxi Datong University, Datong, Shanxi 037009 P. R. China; 5 Department of Urology, MengChao Hepatobiliary Hospital of Fujian Medical University, No.312 Xihong Road, Fuzhou, 350001 China; 6 Department of Ultrasound, Aerospace Center Hospital, Peking University Aerospace School of Clinical Medicine, Beijing, 100049 China; 7 Key Laboratory of Molecular Imaging of Chinese Academy of Sciences, Institute of Automation Chinese Academy of Sciences, Beijing, 100190 P.R. China

**Keywords:** Colorectal cancer, Metastatic lymph nodes, Near-infrared II fluorescence imaging, Molecular imaging, Interferon-induced transmembrane protein 1

## Abstract

**Background:**

To improve the visualisation of colorectal cancer (CRC) and its metastatic lymph nodes (LNs), we developed a second near-infrared window (NIR-II; 1000–1700 nm) fluorescent probe using a monoclonal antibody against interferon-induced transmembrane protein 1 (IFITM1). The NIR-II offers advantages for fluorescence-guided resection of CRC and metastatic LNs.

**Methods:**

IFITM1 expression in CRC was analysed using The Cancer Genome Atlas (TCGA) database. Clinical tumour specimens were assessed by immunohistochemistry to confirm IFITM1 expression in patients with CRC. An IFITM1–IRDye800CW (IFITM1-800CW) NIR-II fluorescent probe was constructed. It’s in vivo performance was evaluated in subcutaneous CRC xenografts, orthotopic implantation models, and orthotopic LN metastasis models. Surgically resected human CRC specimens were also incubated with the probe to evaluate its clinical utility.

**Results:**

TCGA and tissue analyses showed significantly higher IFITM1 expression in CRC compared with normal tissues (*P* < 0·001). After intravenous injection of IFITM1-800CW, tumour fluorescence signals appeared at 6 h and peaked within 24 h across all mouse models, enabling robust CRC and LN visualisation. This approach permitted in situ detection of tumours and metastatic LNs as small as 1 mm. In ex vivo human samples, IFITM1-800CW produced significantly higher fluorescence intensity in CRC tissues than in normal or adjacent tissues (*P* < 0·0001), and allowed identification of tumour margins.

**Conclusions:**

IFITM1 is differentially expressed in CRC and represents a promising imaging target. IFITM1-800CW enables sensitive detection of CRC and metastatic LNs, enabling supporting complete tumour resection under NIR-II fluorescence guidance.

**Supplementary Information:**

The online version contains supplementary material available at 10.1186/s12967-026-07938-0.

## Background

Colorectal cancer (CRC) remains one of the most prevalent malignant tumours worldwide, with high incidence and mortality rates, representing a major public health concern [1–3]. Radical surgical resection is the cornerstone of treatment. However, despite therapeutic advances, the 5-year survival rate of patients with CRC remains approximately 10% [4]. Achieving complete tumour removal (R0 resection) is critical to reducing local recurrence and improving prognosis. Yet, recurrence and metastasis are frequently observed even after R0 resection. Circumferential resection margin (CRM) status is a key prognostic factor in rectal cancer surgery, closely associated with local recurrence and overall survival. Positive tumour margins occur in around 6·0% of cases, with multivariate analyses showing higher rates in advanced stages, particularly in cT4 tumours (up to 14%) [5]. Furthermore, the retrieval of fewer than 12 lymph nodes (LNs) has been linked to increased recurrence risk [6]. Metastasis is the principal driver of recurrence after R0 resection. However, intraoperative assessment often fails to reliably detect metastatic foci, allowing residual disease to persist. Conversely, excessive LN dissection can cause lymphocele formation and adversely affect outcomes [7, 8]. Currently, surgeons rely preoperative imaging modalities and intraoperative visual and tactile assessment, both of which lack precision required for real-time pathological evaluation. As a result, tumour resections may be incomplete, or surgical fields unnecessarily expanded, leading to greater morbidity without improved prognosis.

Intraoperative fluorescence imaging offers a potential solution for accurate real-time detection of tumours and metastatic lesions, thereby enabling complete resection [9]. Fluorescence-guided surgery (FGS) uses targeted fluorescent probes to delineate pathological tissues intraoperatively, improving surgical precision and outcomes [10, 11]. Several fluorescent probes are approved by the US Food and Drug Administration, including 5-aminolevulinic acid (5-ALA) [12] and OTL38 (also known as pafolacianine) [13]. Others, such as bevacizumab-IRDye800CW [14], panitumumab-IRDye800CW [15], and SGM-101 [16], are undergoing clinical evaluation. Indocyanine green (ICG), currently recommended for CRC surgery, accumulates in tumours via passive diffusion and retention mechanisms but lacks tumour specificity. Although ICG can localise LNs, pathological analyses show non-specific uptake in non-lesional regions of metastatic LNs, with poor detection of metastatic sites [17]. Thus, there is an urgent need for novel targeted probes capable of visualising both primary CRC lesions and metastatic LNs during surgery.

Previous studies on FGS have largely employed probes emitting within the visible and first near-infrared window (NIR-I; 700–900 nm). Although these probes have shown utility for vascular imaging and tumour visualisation, their performance is hindered by strong tissue autofluorescence and limited penetration depth at shorter wavelengths [10, 18]. The second near-infrared window (NIR-II; 1000–1700 nm) has recently emerged in preclinical research, offering substantial advantages over NIR-I. NIR-II fluorescence achieves superior resolution, reduced background autofluorescence, higher signal-to-noise ratio, and greater tissue penetration [19, 20]. Preclinical studies have confirmed the strong imaging efficacy of NIR-II FGS in various tumour models [21, 22], highlighting its promise for surgical navigation in CRC. Interferon-induced transmembrane protein 1 (IFITM1) has been reported to be aberrantly overexpressed in CRC while showing negligible expression in normal tissues. Its expression correlates with tumour stage and LN metastasis [23–25]. Beyond its diagnostic value, IFITM1 may contribute to CRC progression through roles in immunomodulation and remodelling of the tumour microenvironment, underscoring its potential as both a biomarker and therapeutic target.

**Fig. 1:**
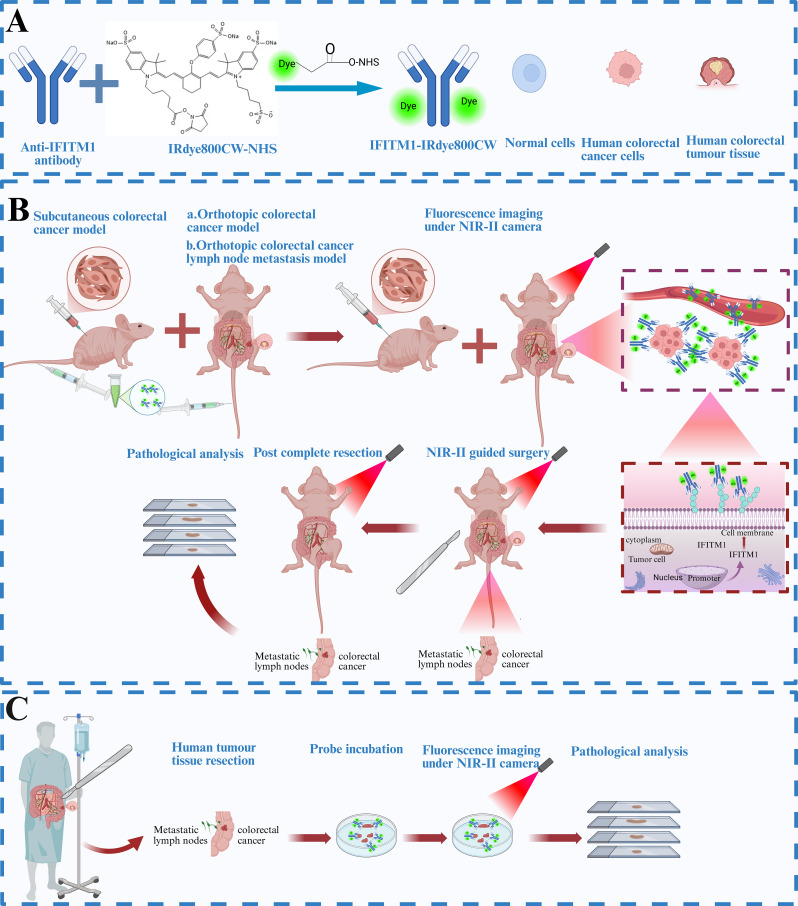
Schematic of IFITM1-IRDye800CW probe design and applications in CRC. (**A**) Synthesis of the IFITM1-IRDye800CW probe and schematic representation of normal cells, CRC cells, and human CRC tissue. (**B**) Establishment of subcutaneous xenografts, orthotopic xenografts, and LN metastasis models in nude mice using human CRC cell lines. Following tail vein injection, the probe binds to IFITM1-overexpressing CRC cells, enabling NIR-II fluorescence-guided resection of primary tumours and metastatic lesions, followed by pathological validation. (**C**) In patients undergoing CRC resection, excised tumour specimen are incubated with IFITM1-IRDye800CW, followed by NIR-II fluorescence imaging and pathological analysis. *Schematic created using the BioRender platform*

In this study, we developed a novel IFITM1-targeted NIR-II fluorescent probe (IFITM1–IRDye800CW; IFITM1-800CW) and evaluated its ability to image CRC lesions both in vitro and in vivo. Our aim was to improve intraoperative tumour delineation, reduce the rate of positive margins, and enable reliable detection of metastatic LNs (Fig. 1), thereby supporting surgeons in achieving comprehensive and precise resections.

## Methods

### Ethics statement

Clinical tissue specimens study were obtained with verbal informed consent from all patients. The study was approved by the Ethics Committee of Fujian Cancer Hospital (SQ2021-172-01). All animal experiments were approved by the Institutional Animal Care and Use Committee of Fujian Medical University and conducted in accordance with institutional guidelines for the care and use of laboratory animals.

### IFITM1 expression in CRC tissue

RNA sequencing (RNA-Seq) data were obtained from The Cancer Genome Atlas (TCGA) database (https://portal.gdc.cancer.gov). RNA-Seq data from the TCGA-COAD and TCGA-READ projects in STAR format were downloaded and processed into transcripts per million (TPM) format. Paired-end RNA-Seq data from cancer-adjacent tissue (*n* = 50) and tumour samples (*n* = 50) were analysed to compare IFITM1 mRNA expression in CRC and normal tissue pairs. In addition, RNA-Seq data from 647 CRC and 51 normal tissue samples were extracted from TCGA to further assess IFITM1 mRNA expression and to evaluate its predictive accuracy. The association between IFITM1 expression and CRC stage was examined using the GEPIA database (http://gepia2.cancer-pku.cn/#/index). Thirty-six clinical CRC specimens were also collected for IFITM1 protein expression analysis.

### Immunohistochemistry (IHC)

Paraffin-embedded sections were mounted on slides, cooled to room temperature, dewaxed, and rehydrated using xylene and graded ethanol (100%, 95%, and 75%). Antigen retrieval was performed in citrate buffer using a high-pressure thermal fixator. Sections were blocked with 5% normal goat serum and incubated overnight at 4 °C with a primary antibody against IFITM1 (ab255273, 1:500; Abcam, Cambridge, UK). After washing, sections were incubated with a secondary antibody (SA00001-2, 1:2000; Proteintech, Wuhan, China), followed by 3,3′-diaminobenzidine (DAB) staining and haematoxylin counterstaining. Images were acquired using an Aperio GT 450 scanner (Leica Biosystems). Pathological evaluation was performed with ImageJ IHC Profiler software (National Institutes of Health, USA), with IFITM1 overexpression defined as staining intensity greater than 50%.

### Synthesis and characterisation of mAb-IFITM1-IRDye800CW

To construct the synthetic NIR-II fluorescent probe, an IFITM1 monoclonal antibody (mAb; ab255273; Abcam, UK) was used as the ligand. The NIR dye IRDye800CW-NHS ester (LI-COR Biosciences) was selected for labelling owing to its favourable biosafety profile Probe synthesis followed the method described for EGFR–IRDye800CW [26]. Briefly, the antibody was dialysed in 0·01 M phosphate-buffered saline (PBS), adjusted to pH 8·5 with 0·05 M NaHCO₃. IRDye800CW-NHS dissolved in dimethyl sulfoxide (DMSO) (10 µg/µL) was added to the protein solution and allowed to react in the dark for 2 h. The mixture was centrifuged 12,000 rpm to remove denatured proteins, and free dye was removed by dialysis, yielding the purified probe. For probe characterization, absorbance at 280 nm and 774 nm was measured using a UV spectrophotometer, and dye-to-antibody coupling ratio was calculated. Absorption and emission spectra were obtained to establish the relationship between probe concentration and fluorescence intensity. Particle size was measured using a dynamic light scattering (DLS) analyser.

### Cell culture

Human CRC cell lines NCM40 (RRID: CVCL_0460), SW480-Luc (RRID: CVCL 0546), SW620 (RRID: CVCL_0547), HT29 (RRID: CVCL_0320), and RKO (RRID: CVCL_0504) were purchased from Zhejiang Mason Cell Technology Co. Cells were cultured in Dulbecco’s Modified Eagle Medium (DMEM; 11965092; Gibco, Thermo Fisher Scientific, USA) supplemented with 10% foetal bovine serum (A5256701; Gibco, Thermo Fisher Scientific, USA) and 1% penicillin–streptomycin (Servicebio, Wuhan, China). Cultures were maintained at 37 °C in a humidified atmosphere containing 5% CO₂ using a constant-temperature incubator.

### Western blotting (WB)

Cells were harvested, and membrane proteins were extracted using a membrane protein extraction kit (G2280-50T; Servicebio, Wuhan, China). Protein concentrations were measured with a BCA protein assay kit (P0398M; Biyun Tian, Shanghai, China). Samples were denatured at room temperature, and 40 µg of membrane protein was loaded onto a 12·5% sodium dodecyl sulphate polyacrylamide gel (SDS-PAGE). Following electrophoresis, proteins were transferred onto a 0·22 μm PVDF membrane and blocked with 5% skimmed milk at room temperature. Membranes were incubated overnight at 4 °C with rabbit anti-IFITM1 antibody (ab255273, 1:1000; Abcam, UK) and anti-β-actin antibody (66009-1-Ig, 1:20,000; Proteintech, Wuhan, China). After washing with Tris-buffered saline Tween (TBST), membranes were incubated with anti-rabbit secondary antibody (SA00001-2, 1:2000; Proteintech, Wuhan, China) or anti-mouse secondary antibody (SA00001-1, 1:2000; Proteintech, Wuhan, China) at room temperature. Protein bands were visualised using enhanced chemiluminescence on an Image Lab™ system (Bio-Rad) and quantified using ImageJ software after background subtraction, with normalisation to β-actin as the loading control.

### In vitro targeting evaluation

SW480-Luc, HT29 and RKO tumour cells were seeded at 1–5 × 10⁵ cells/mL into 15 mm glass-bottom confocal dishes. After 24 h, 200 µL of IFITM1-IRDye800CW (10 µg/mL) was added and incubated in the dark for 1 h. Cells were then fixed with 4% paraformaldehyde, incubated with goat anti-rabbit IgG H&L (Alexa Fluor^®^ 488; 10 µg/mL, ab150077; Abcam, UK) for 30 min, and stained with 1,1′-Dioctadecyl-3,3,3′,3′-tetramethylindocarbocyanine (DiI, D282; Thermo Fisher Scientific, USA) for cell membrane colocalisation. Nuclei were counterstained with 4′,6-diamidino-2-phenylindole (DAPI) before imaging with a laser scanning confocal microscope (LSM780; Carl Zeiss, Jena, Germany). For binding-site competition assays, free IFITM1 antibody was added at 100-fold excess relative to the probe concentration and incubated with cells for 3 h. Thereafter, the probe was added, and cells were fixed with 4% paraformaldehyde. Subsequent staining with goat anti-rabbit IgG H&L (Alexa Fluor^®^ 488), DiI, and DAPI was performed as described above, followed by confocal microscopy.

### Human CRC cell line xenograft model

Five-week-old male BALB/c-nu mice were purchased from Guangdong YaoKang Biotechnology Co., Ltd. SW480-Luc cells (high IFITM1 expression), RKO cells (moderate expression), and SW620 cells (low IFITM1 expression) were cultured and prepared for xenografting. For subcutaneous xenografts, 2 × 10⁶cells were inoculated into the right flank of BALB/c-nu mice. Tumours were harvested for imaging once they reached approximately 1 cm in diameter. Orthotopic colonic tumour and LN metastasis models were established by midline laparotomy. Using a sterile 40G insulin syringe (0·3 mm × 8 mm) 1 × 10⁶ SW480-Luc cells in suspension were slowly injected into the serosal layer of the caecum, generating orthotopic CRC and LN metastasis models in nude mice. Tumour formation and progression were monitored periodically using in vivo small-animal imaging.

### NIR-I/II fluorescence imaging

The NIR-I imaging system comprised a high-sensitivity complementary metal–oxide–semiconductor (CMOS) camera (PCO.edge 5·5 m; PCO AG, Germany) fitted with a lens (EF 24–70 mm f/2·8 L II USM; Canon, Japan) and a bandpass filter (840 ± 40 nm BP; FF01-832/37, Semrock, USA) coupled via an adapter. Excitation was provided by 785 nm and 808 nm lasers (MW-GX-785 and MW-GX-808), with exposure time set at 300 ms. The NIR-II imaging system consisted of a compact high-resolution near-infrared camera (Cheetah-640CL TE3; Xenics, Belgium) equipped with a low-noise InGaAs detector array (0·9–1·7 μm) and paired with a shortwave infrared lens (VF50M SWIR, 50 mm focal length; Spacecom, Japan). Fluorescence was captured using filters at 900, 1000, 1100, 1200, 1300, 1400, and 1500 nm (Thorlabs, USA). Excitation was provided by an 808 nm laser at 25 mW/cm², with exposure times ranging from 10 ms to 1500 ms.

### In vivo fluorescence imaging

Based on quantitative western blot analysis, subcutaneous CRC models were established in nude mice using cell lines with low, medium, and high IFITM1 expression. Each group contained at least three mice. An additional three bearing the high-IFITM1-expressing xenografts were assigned as the experimental control group. Tumour size was monitored and measured intermittently. The probe was administered via tail vein injection at a dose of 2 µg/g of IFITM1-800CW. Control mice received an intravenous injection of IFITM1 antibody at a 100-fold excess relative to probe concentration 24 h before probe administration to block IFITM1 binding, followed by injection of IFITM1-800CW. Imaging was performed at 1, 6, 12, 24, 48, and 72 h post-injection to assess tumour visualisation over time. Fluorescence images were pseudo-coloured and quantified using ImageJ, and the tumour-to-background ratio (TBR) was calculated as the mean fluorescence intensity of tumour tissue divided by that of adjacent skin. At 72 h, mice were euthanised, and tumours and major organs were collected for examination under white light and NIR-II imaging.

### NIR-II fluorescence-guided CRC resection

Three mice bearing orthotopic SW480-Luc tumours underwent preoperative magnetic resonance imaging (MRI; Bio Spec 94/30 USR, Bruker, Germany) for non-invasive assessment of tumour volume and localisation. All mice received IFITM1-800CW by intravenous injection 24 h before surgery. Surgical procedures were performed under tribromoethanol anaesthesia (T48402-25G; Sigma-Aldrich, Merck, Darmstadt, Germany). White light, NIR-I, and NIR-II illumination were sequentially applied during surgery, and tumour resection was carried out under NIR-II fluorescence guidance. Tumour clearance was confirmed by bioluminescence imaging (BLI) using an IVIS Spectrum system (PerkinElmer, Waltham, MA, USA). Intraoperative fluorescence intensity (grey value) was measured at the maximum tumour cross-section to compare the imaging efficacy of NIR-I versus NIR-II. After resection, tumours and anatomical organs were examined under white light and NIR-II to evaluate probe distribution. Tissue samples were then subjected to histopathological analysis by haematoxylin and eosin (H&E) staining to verify negative tumour margins.

For LN metastasis models, three mice bearing orthotopic SW480-Luc tumours with LN metastases were injected with IFITM1-800CW 24 h before surgery. Procedures were performed under tribromoethanol anaesthesia., White light and NIR-II fluorescence were applied intraoperatively. As mesenteric LN metastases were not clearly visualised in vivo, the intestines were repositioned on black cardboard to enhance signal detection. Tumours and metastatic LNs were excised under combined white light and NIR-II guidance. Postoperatively, ex vivo NIR-II fluorescence imaging was used to assess tumours and metastatic LNs. Histopathological confirmation was performed using H&E staining and IHC.

### Fresh human CRC specimens for in vitro molecular imaging

Colon and rectal cancer specimens (*n* = 20) were collected from surgical resections performed at the Department of Colorectal Surgery, Fujian Cancer Hospital. Fresh tissues were washed with PBS to remove surface blood, immersed in IFITM1-800CW probe solution (10 µg/mL), and incubated in the dark for 30 min. Samples were then washed with PBS and imaged using an NIR-II camera. Histopathological features were confirmed by H&E staining, and IFITM1 expression was verified by IHC.

### Statistical analysis

Data are expressed as mean ± standard deviation (SD). Differences between two groups were analysed using Student’s *t-*test. When multiple groups satisfy the normality assumption, repeated measures analysis of variance (ANOVA) is employed for testing, with results adjusted using the Greenhouse-Geisser correction. Analyses were conducted using GraphPad Prism 10 (GraphPad Software, USA) and Origin 2024b (OriginLab, USA). The immunohistochemical H-score was calculated as H = ∑(pi × i), where *pi* represents the percentage of positive cells at a given intensity, and *i* represents the staining intensity grade: 0 (negative, no staining), 1 (weak, pale yellow), 2 (moderate, brownish-yellow), and 3 (strong, dark brown). The formula is expressed as: (percentage of weakly positive cells × 1) + (percentage of moderately positive cells × 2) + (percentage of strongly positive cells × 3).

Quantitative analysis of NIR fluorescence images and IHC results was performed using ImageJ software, with data converted to pseudocolour. A *P* value < 0·05 was considered statistically significant. Statistical significance was denoted as follows: *P < 0·05; **P < 0·01; ***P < 0·001; ****P < 0·0001.

### Role of the funding source

The sponsors of the study had no role in the study design, data collection, data analyses, data interpretation, manuscript writing, or the decision to submit the paper for publication.

## Results

### IFITM1 expression in CRC tissue

**Fig. 2:**
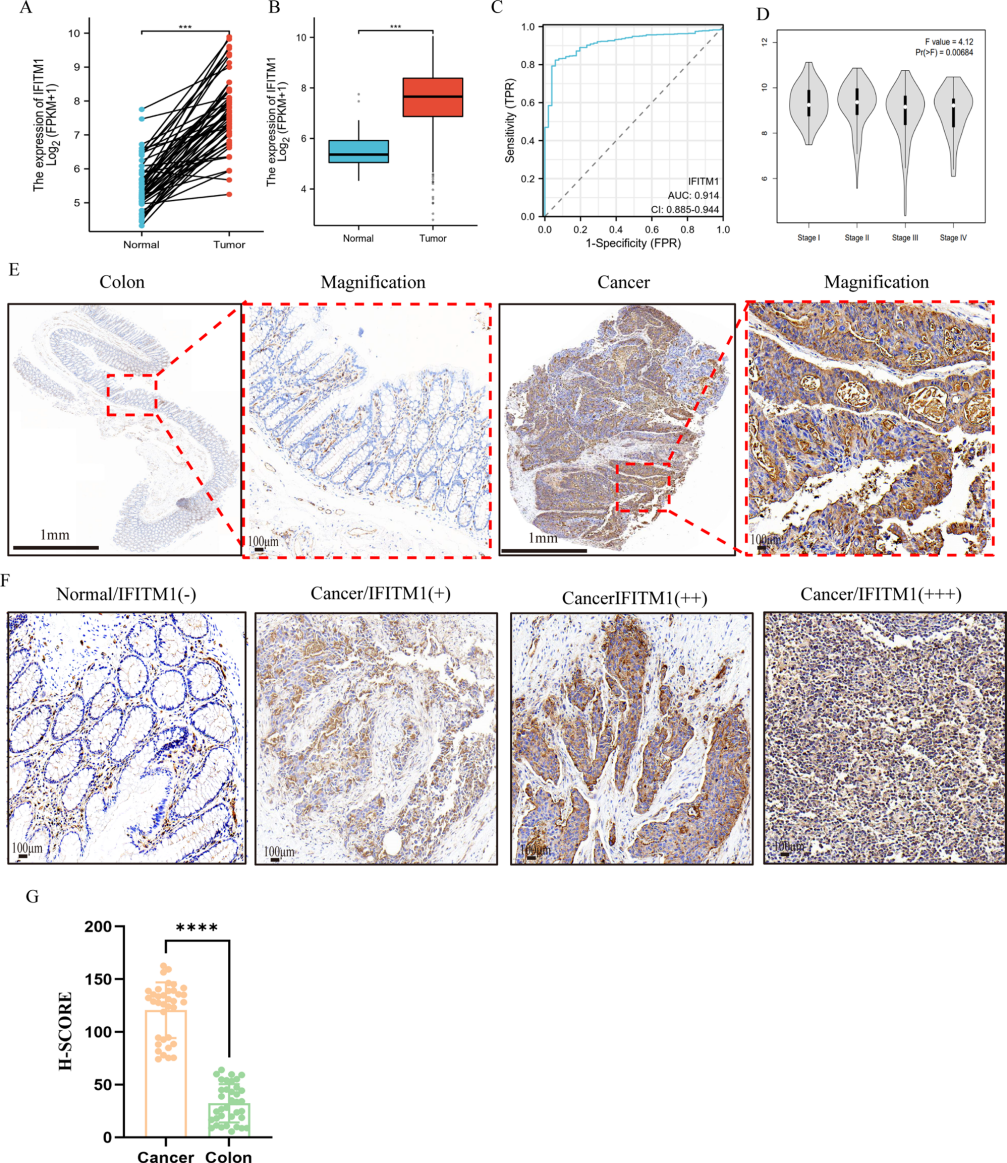
Expression of IFITM1 in normal intestinal mucosa and CRC tissue. (**A**) IFITM1 expression in paired cancer-adjacent (*n* = 50 ) and tumour (*n* = 50) samples analysed from the TCGA database. (**B**) IFITM1 expression in CRC (*n* = 647) versus normal tissue (*n* = 51) from the TCGA database. (**C**) ROC analysis of IFITM1 using TCGA data. (**D**) Correlation between IFITM1 expression and CRC stage based on the GEPIA database. (**E**) IHC staining of IFITM1 in normal intestinal mucosa and CRC tissues. (**F**) Representative IHC images showing differential expression of IFITM1 in CRC versus normal mucosa. (**G**) Quantitative analysis of IFITM1 expression (H-score) in CRC tissues (*n* = 36 ) compared with normal mucosa (*n* = 36). ***P < 0·001, ****P < 0·0001. Scale bar: 1 mm, 100 μm

To validate IFITM1 protein expression, paired cancer-adjacent (*n* = 50) and tumour (*n* = 50) samples were extracted from the TCGA database. IFITM1 expression was significantly higher in tumour tissues compared with matched cancer-adjacent tissues (*P* < 0·001; Fig. 2A). RNA-Seq data from 647 CRC and 51 normal tissue samples in TCGA confirmed elevated IFITM1 expression in CRC relative to normal tissues (*P* < 0·001; Fig. 2B). Receiver operating characteristic (ROC) curve analysis yielded an area under the curve (AUC) of 0·914, indicating high predictive accuracy for CRC (Fig. 2C). Analysis using the GEPIA database further revealed a positive correlation between IFITM1 expression and CRC stage (Fig. 2D). Histological analysis demonstrated low IFITM1 expression in normal intestinal mucosa but strong expression in CRC tissues (Fig. 2E). IHC categorised IFITM1 expression as negative (−), weak (+), moderate (++), and strong (+++). Although IFITM1 was expressed in all tumour tissue samples, heterogeneity across tumour regions were observed. Some CRC tissues exhibited weak staining, whereas normal tissues showed only faint expression, which was significantly lower than that in weakly stained tumour areas (Fig. 2F). Compared with normal intestinal mucosa, CRC tissues displayed significantly higher H-scores for IFITM1 expression (*P* < 0·0001; Fig. 2G).

### Fluorescence characterisation of IFITM1-IRDye800CW

**Fig. 3:**
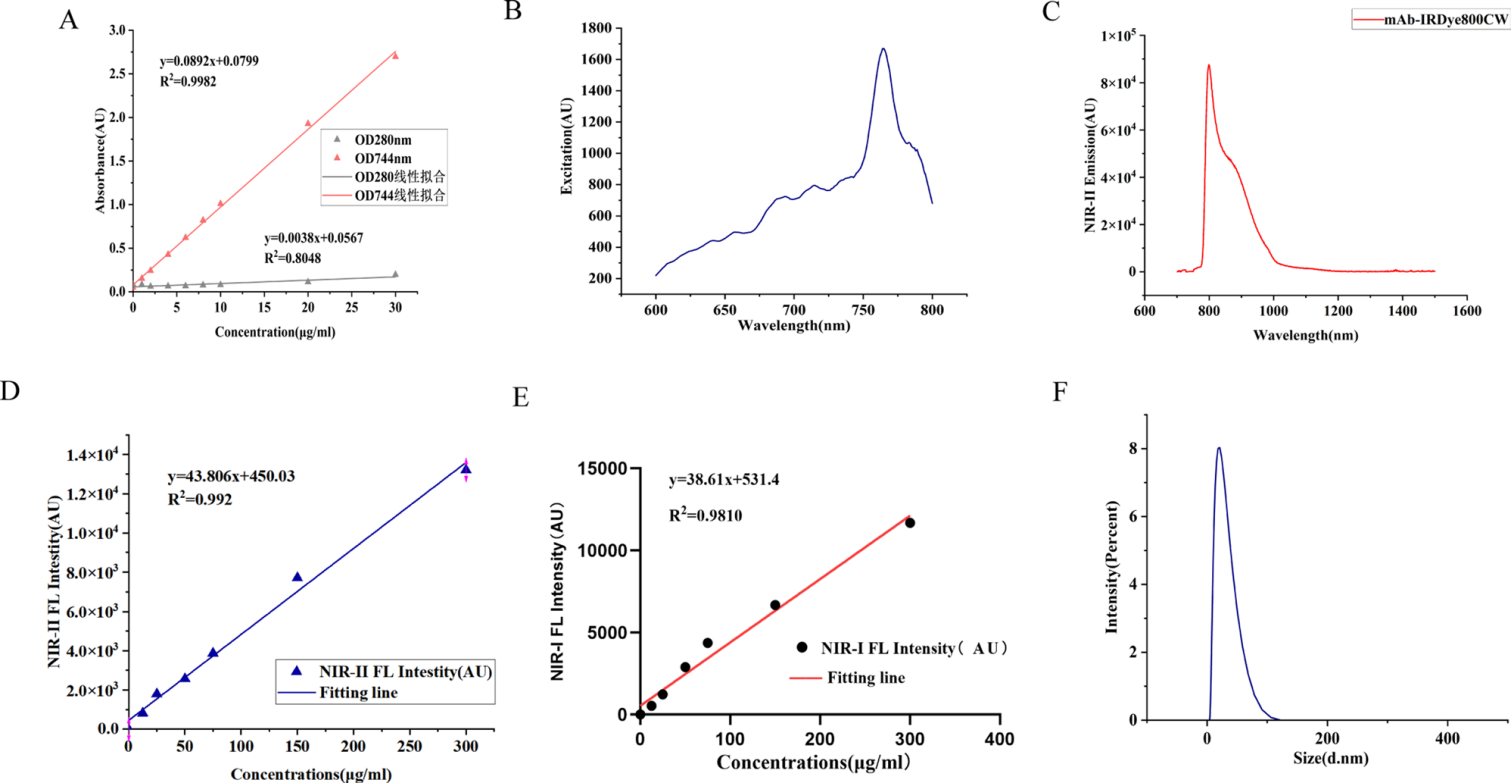
Characterisation of the IFITM1-800CW NIR-II fluorescent probe. (**A**) Standard curve of probe absorbance values at 280 nm and 774 nm measured by ultraviolet spectrophotometry. (**B**) Excitation spectrum of IFITM1-800CW. (**C**) Emission spectrum of IFITM1-800CW. (**D**) Scatter plot with linear fit showing correlation between probe concentration and NIR-II fluorescence intensity (R² = 0·992). (**E**) Scatter plot with linear fit showing correlation between probe concentration and NIR-I fluorescence intensity (R² = 0·9810). (F) Hydrodynamic particle size of IFITM1-IRDye800CW determined by DLS

The synthesised probe was diluted into a series of concentration gradients, and absorbance at 280 nm and 774 nm was measured to generate a standard curve (Fig. 3A). The fluorophore-to-protein (F/P) ratio for IFITM1-800CW was calculated as 2·1, indicating an average of 2·1 dye molecules conjugated per antibody. Spectral analysis showed an absorption peak at 775 nm and an emission peak at 799 nm. (Fig. 3B–C). The probe produced strong fluorescence signals across the 900–1500 nm NIR-I range. At constant concentration, the fluorescence intensity of IFITM1-800CW demonstrated a linear relationship with the probe concentration in both the NIR-II window (R² = 0·992, Fig. 3D) and the NIR-I window (R² = 0·9810, Fig. 3E). DLS analysis determined a hydrodynamic particle size of 21 nm (Fig. 3F). Collectively, these findings confirm the successful synthesis of a stable optical probe with high labelling efficiency and robust fluorescence properties.

### Expression of IFITM1 in human CRC cell lines and in vitro binding capacity of IFITM1-IRDye800CW

**Fig. 4:**
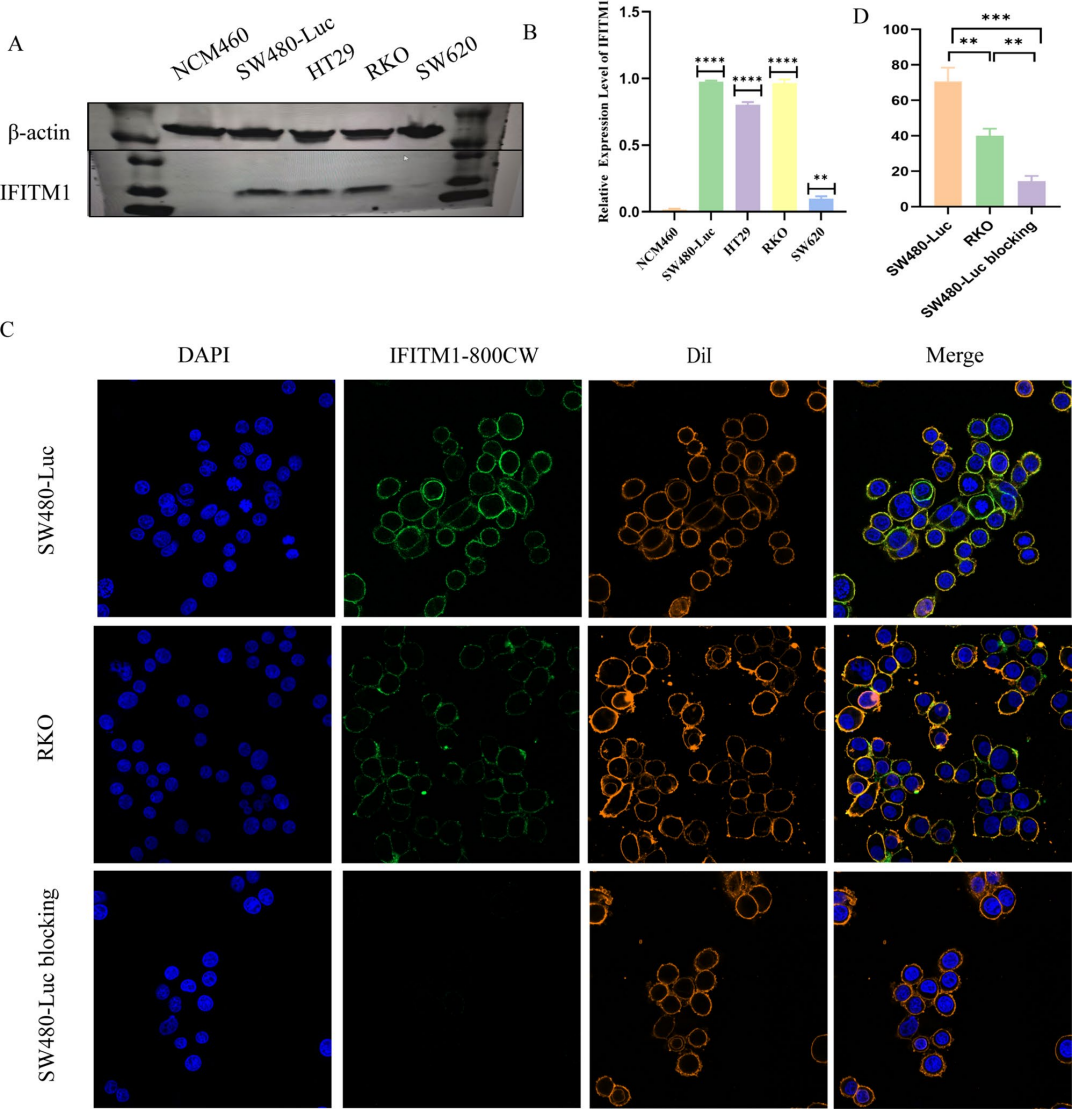
Expression of IFITM1 in CRC cell lines and binding capacity of IFITM1-800CW. (**A**) Western blot analysis of IFITM1 in different CRC cell lines. (**B**) Quantitative analysis of IFITM1 expression based on western blot results. (**C**) Co-localisation of IFITM1-800CW (green) with the membrane dye DiI (orange) in SW480-Luc, RKO, and SW480-Luc blocking group cells; nuclei stained with DAPI (blue). (**D**) Statistical analysis of fluorescence intensity following probe incubation across all cell groups. **P < 0·01, ***P < 0·001, ****P < 0.0001. Magnification: 40⋅

Four human CRC cell lines (SW480-Luc, SW620, HT29, and RKO) and the normal human colonic mucosal epithelial cell line NCM460 were cultured, and IFITM1 expression was assessed. Western blot analysis showed IFITM1 overexpression in all tumour cell lines, whereas NCM460 cells exhibited negligible expression (Fig. 4A–B). Quantitative analysis of IFITM1/β-actin levels revealed significantly higher expression in SW480-Luc cells compared with HT29 and RKO cells. Consequently, SW480-Luc cell line was selected as the primary study model.

To evaluate the in vitro targeting ability of IFITM1-IRDye800CW, SW480-Luc (high expression) and RKO (moderate expression) cells were incubated with 0·01 µg/mL probe for 3 h. Confocal microscopy revealed strong green fluorescence signals in SW480-Luc cells (Fig. 4C). Co-localisation with DiI membrane staining (red) confirmed that probe-derived green fluorescence overlapped with the red membrane signal. In contrast, RKO cells (moderate expression) exhibited weaker probe fluorescence. Quantitative analysis demonstrated significantly higher fluorescence intensity in SW480-Luc cells compared with RKO cells (*P* = 0·001; Fig. 4C-D). To further verify probe specificity, a blocking assay was performed in SW480-Luc cells. Pre-treatment with excess IFITM1 mAb markedly reduced probe binding, resulting in only faint fluorescence, whereas cells incubated directly with IFITM1-IRDye800CW exhibited strong fluorescence signals (Fig. 4C-D). Statistical analysis confirmed a significant reduction in probe fluorescence intensity in the blocking group compared with unblocked SW480-Luc cells (*P* < 0·001; Fig. 4C-D). These results indicate strong binding affinity and specificity of IFITM1-IRDye800CW for IFITM1-overexpressing CRC cells.

### In vivo biodistribution and targeting of the IFITM1-IRDye800CW

**Fig. 5:**
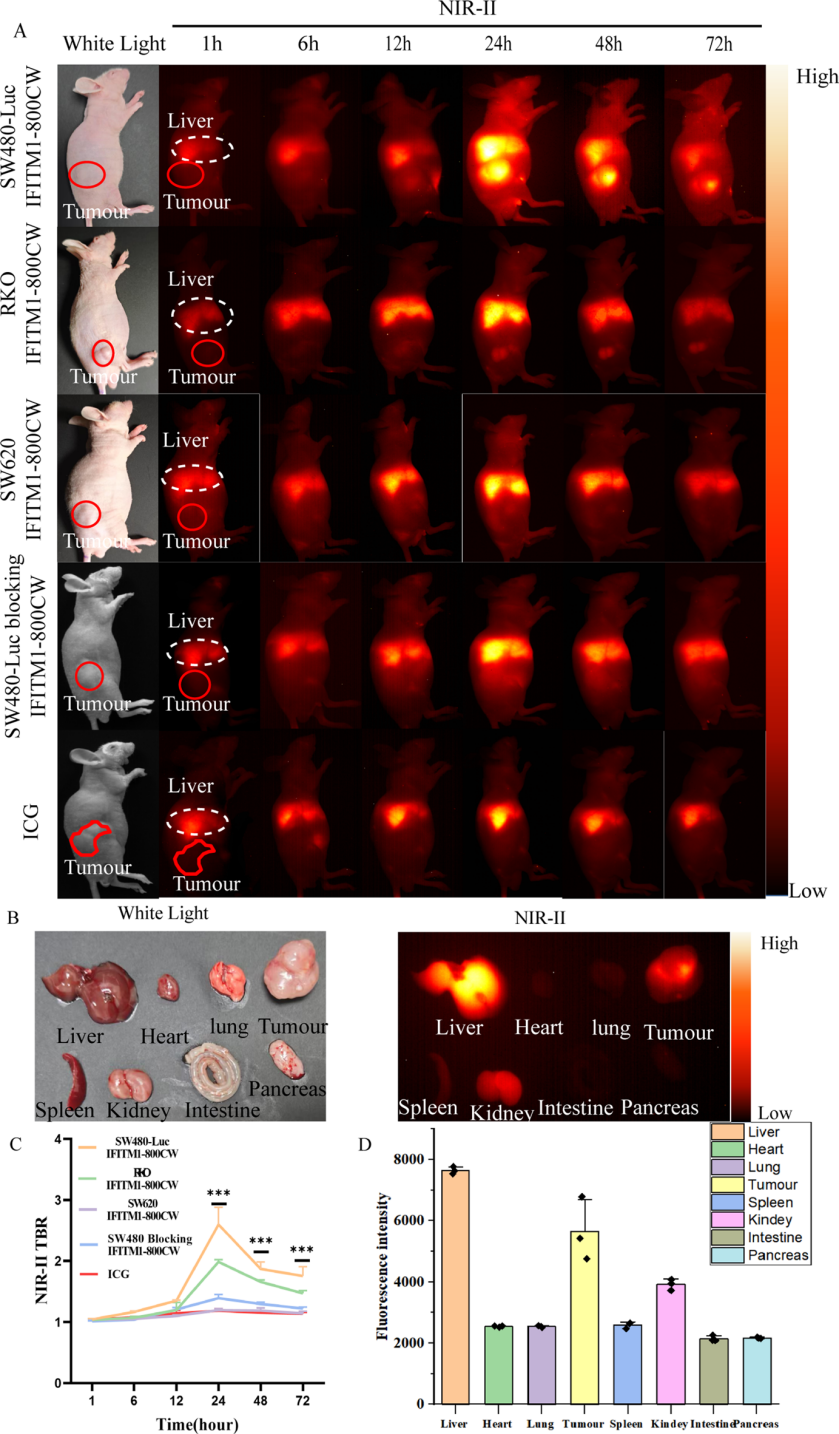
Evaluation of IFITM1-IRDye800CW specificity and biodistribution in vivo under NIR-II imaging. (**A**) White light imaging of SW480-Luc, RKO, and SW620 experimental groups alongside SW480-Luc control group; NIR-II fluorescence imaging of these groups following IFITM1-IRDye800CW injection at 1, 6, 12, 24, 48, and 72 h post-injection. (**B**) 24 h post-probe injection, organs and tumour tissues were imaged under white light and NIR-II. (**C**) Analysis the trend in TBR changes over 1–72 h between the SW480-Luc, RKO and SW620 experimental groups and the SW480-Luc control group, with between-group comparison of TBR. Fluorescence signal from the skin surrounding the tumour was selected as the background reference. (**D**) Statistical analysis of organ fluorescence intensity. Statistical analysis (t-test): **P < 0.01, ***P < 0.001

Based on western blot analysis, subcutaneous tumour models were classified as IFITM1-high (SW480-Luc), IFITM1-moderate (RKO), IFITM1-low (SW620), and IFITM1-high with blocking (SW480-Luc pretreated with free IFITM1 antibody). When tumours reached approximately 1 cm, IFITM1-IRDye800CW was administered via tail vein injection. NIR-II imaging was performed at 1, 6, 12, 24, 36, 48 h, and 72 h to evaluate tumour visualisation and probe biodistribution (Fig. 5A). In SW480-Luc mice (IFITM1-high, *n* = 3), strong fluorescence signals were initially observed in the liver, consistent with hepatic metabolism of IFITM1 antibodies. Fluorescence gradually diminished but tumour signals became detectable at 6 h, peaked at 24 h (TBR = 2·59 ± 0·28), and persisted up to 72 h (Fig. 5A, C). In RKO mice (IFITM1-moderate, *n* = 3), which moderately expressed IFITM1, tumour fluorescence peaked at 24 h (TBR = 1·99 ± 0·37; *P* < 0·001) but attenuated rapidly, remaining significantly weaker than in SW480-Luc mice (Fig. 5A, C). In SW620 mice (IFITM1-low, *n* = 3), tumour fluorescence was essentially absent, with a maximum TBR of 1·20 ± 0·07 (*P* < 0·001; Fig. 5A, C). In the SW480-Luc blocking group (IFITM1-high, *n* = 3), pre-administration of excess IFITM1 antibody via tail vein injection 24 h prior to probe delivery markedly reduced probe uptake. Tumour fluorescence intensity was faint, with a maximum TBR of 1·39 ± 0·14 (*P* < 0·001; Fig. 5A, C), confirming receptor-specific targeting of the probe. At the same time, ICG solution with the same concentration and volume as the IFITM1-800CW probe was injected via the tail vein. The results showed that at 24 h, the IFITM1-800CW probe still maintained a high TBR (2.59 ± 0.57), while ICG was basically cleared (TBR = 1.18 ± 0.05, *p* < 0.001, Figure S1). E*x vivo* NIR-II imaging of dissected organs and tumours from SW480-Luc mice revealed probe accumulation predominantly in the liver and tumour tissues, with only faint signals in the spleen and kidney (Fig. 5B, D). Together, these findings demonstrate that IFITM1-IRDye800CW specifically targets CRC cells in vivo, with fluorescence intensity correlating with IFITM1 expression levels.

### NIR-II fluorescence imaging-guided orthotopic tumour resection

**Fig. 6:**
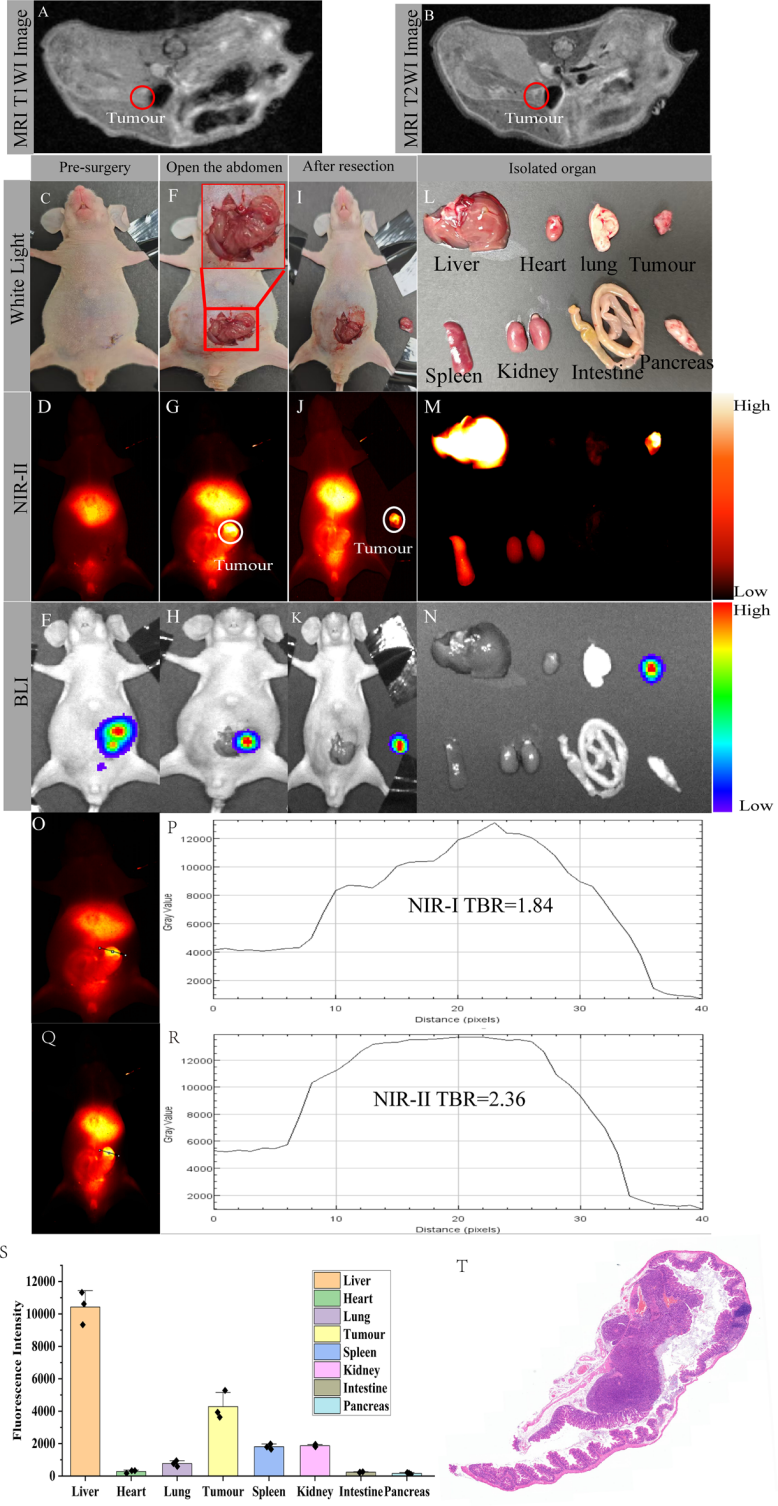
NIR-II fluorescence-guided CRC resection. (**A**) MRI T1-weighted image of an orthotopic tumor-bearing mouse. (**B**) MRI T2-weighted image of an orthotopic tumor-bearing mouse. (**C**-**E**) Following tail vein injection of the probe, orthotopic colorectal tumours in mice (*n* = 3) were imaged under white light, NIR-II, and BLI, respectively. (**F**) After laparotomy, the orthotopic tumour was visualized under white light to assess its size and location; the tumour size is shown at higher magnification in the red box. (**G**) Following laparotomy, the size, location, and boundaries of the orthotopic tumour relative to the surrounding tissues were observed under NIR-II fluorescence. (**H**) The tumour location identified by BLI imaging was consistent with that observed under white light and NIR-II fluorescence. (**I**) White light imaging of the excised tumour. (**J**) The excised tumour was imaged under NIR-II fluorescence, and the abdominal cavity was examined for residual fluorescence signals. (**K**) The excised tumour was imaged using BLI, and the abdominal cavity was examined for residual tumour fluorescence signals. (**L**-**N**) Following excision, the major organs and tumours were subjected to imaging under white light, NIR-II, and BLI, and the variations in fluorescence intensity were subsequently analysed. (**O**) Cross-sectional analysis of orthotopic tumour in NIR-I. (**P**) Cross-sectional analysis of orthotopic tumour in NIR-I showing TBR values. (**Q**) Cross-sectional analysis of the orthotopic tumour in NIR-II. (**R**) Cross-sectional analysis of the orthotopic tumour in NIR-II showing TBR values. (i) Ratio of NIR-I to NIR-II fluorescence intensity for tumour. (**S**) Statistical analysis of organ fluorescence intensity. (**T**) H&E staining of the excised tumour. Scale bar: 1000 μm

To recapitulate the clinical setting of CRC, an orthotopic xenograft model of the caecum was established using SW480-Luc cells (*n* = 3), which exhibit high IFITM1 expression. Cells were implanted into the serosal layer of the mouse caecum. MRI confirmed orthotopic tumour formation, localisation, and size (Fig. 6A–B). Twenty-four hours after intravenous injection of IFITM1-IRDye800CW, with fasting during this period, tumours were resected under combined white light and NIR-II fluorescence guidance. Tumour localisation was validated by bioluminescence imaging (BLI). Before laparotomy, tumour location and size were indistinguishable under both white light (Fig. 6C) and NIR-II fluorescence (Fig. 6D), whereas the liver exhibited strong fluorescence signals. BLI subsequently confirmed tumour localisation (Fig. 6E). After disinfection, the abdominal wall was opened to expose the caecum. Under white light, the tumour tissue was difficult to distinguish from surrounding normal tissue (Fig. 6F). In contrast, NIR-II fluorescence clearly delineated the tumour, which showed stronger signals than adjacent tissue (Fig. 6G). BLI corroborated tumour burden signals (Fig. 6H). By overlaying BLI and NIR-II fluorescence, nodules within overlapping regions of high signal were identified as tumours. Tumour margins remained indistinct under white light (Fig. 6I), but were clearly defined under NIR-II imaging (Fig. 6G). Guided by NIR-II fluorescence, lesion tissues were excised completely. Post-resection evaluation under both modalities revealed no residual fluorescence at the tumour site (Fig. 6J), and BLI confirmed the absence of residual tumour burden (Fig. 6K). Comparison of probe performance revealed that NIR-II fluorescence provided stronger contrast than NIR-I, with TBRs of 2·36 for NIR-II versus 1·84 for NIR-I (Fig. 6O–R). Ex vivo analysis of organs under white light, NIR-II, and BLI confirmed probe accumulation predominantly in the liver (Fig. 6L–N and S). Histopathological analysis of excised tumours by H&E staining confirmed negative resection margins (Fig. 6T). We analyzed the differences in tumour imaging between the NIR-II and NIR-I in three orthotopic mice. The results showed that the NIR-II window exhibited a higher TBR (2.61 ± 0.48 vs. 1.77 ± 0.16, *P* = 0.0447, t-test, Figure S2).This findings demonstrate that IFITM1-IRDye800CW enables precise intraoperative localisation and complete R0 resection of orthotopic CRC tumours under NIR-II fluorescence guidance.

### Fluorescence imaging of orthotopic CRC–metastatic LNs

**Fig. 7:**
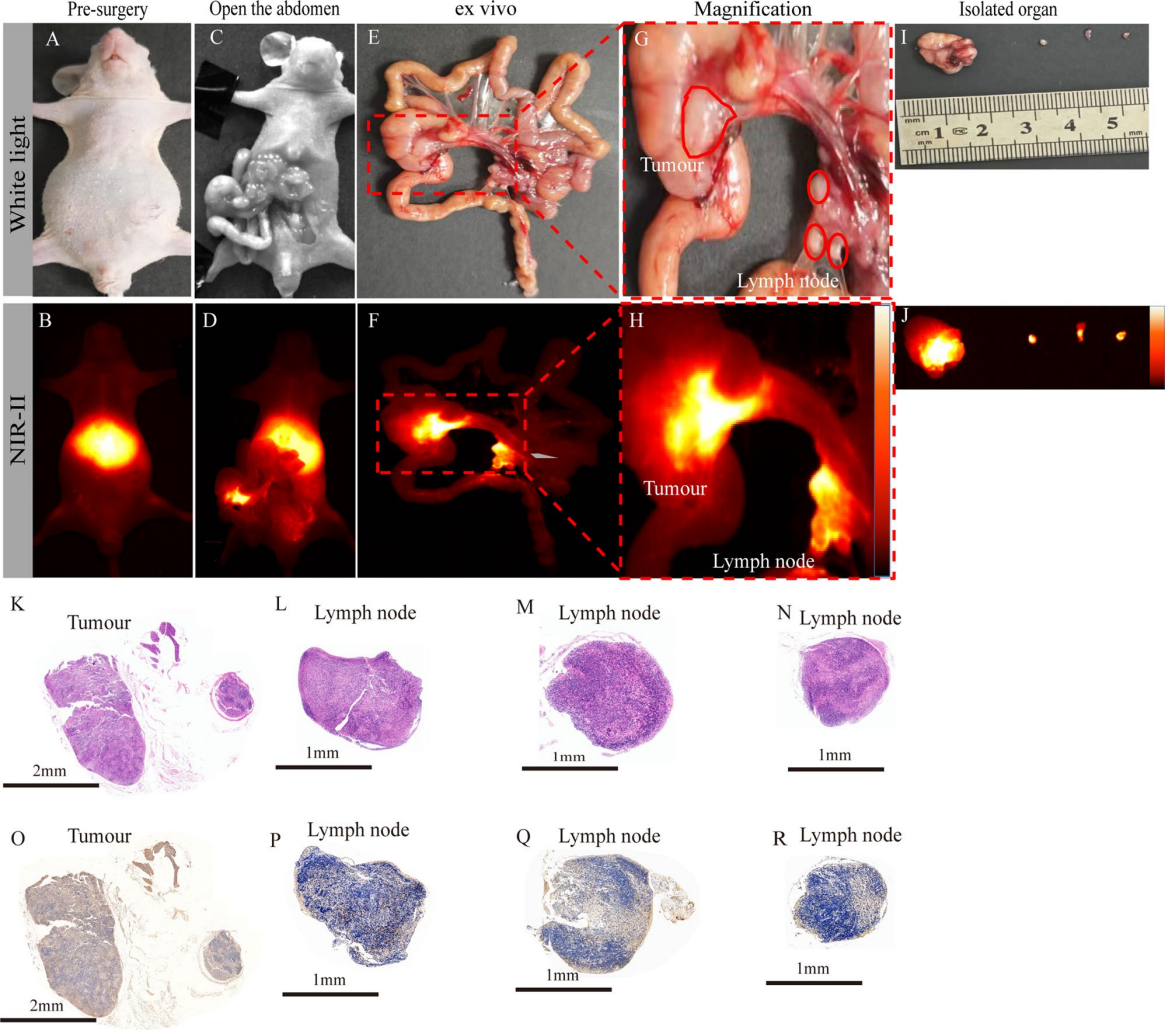
Near-infrared II fluorescence-guided resection of colorectal cancer and its lymph nodes. (**A**–**B**) Preoperative observation under white light and NIR-II fluorescence. (**C**–**D**) Intraoperative white light and NIR-II fluorescence following laparotomy.Visual inspection under both white light and NIR-II fluorescence intraoperatively following laparotomy. (**E**–**F**) Due to the inability to fully unfurl the mesentery, the entire intestinal tract was excised and arranged in its physiological sequence. Imaging was then performed under both white light and NIR-II fluorescence. (**G**–**H**) Magnified views of the tumour and its metastatic lymph node regions, delineated with red outlines. (**I**–**J**) Postoperative tumour and LN specimens under white light and NIR-II fluorescence. (**K**–**R**) Postoperative histological analysis of tumours and LNs by HE and IHC. Scale bar: 1000 μm

The specificity of IFITM1-IRDye800CW was further validated using an orthotopic human tumour model, as LN metastasis represents the most common route of local spread in CRC. To evaluate its diagnostic performance for LN metastasis, a orthotopic CRC model that develops LN metastases was established by injecting SW480-Luc cells into the serosal layer of the mouse caecum (*n* = 3). After tail vein administration of IFITM1-IRDye800CW, white light imaging revealed abdominal tumours and metastatic LNs (Fig. 7A). At 24 h, NIR-II imaging showed strong liver fluorescence but no detectable tumour signal before laparotomy (Fig. 7B). Following laparotomy, the colorectal carcinoma became visible in situ under white light (Fig. 7C). In contrast, NIR-II fluorescence clearly delineated the carcinoma, with tumour signal intensity significantly higher than surrounding tissues (Fig. 7D). Because the mesentery remained folded, metastatic LNs were not visualised in vivo. After euthanasia, the intestine was excised intact and arranged in a physiological sequence on black cardboard, enabling identification of primary tumour and metastatic LNs within the mesentery (Fig. 7E, G; red circles). Under NIR-II imaging, both the orthotopic tumour and metastatic LNs exhibited markedly stronger fluorescence than surrounding tissues (Fig. 7F, H). Sequential resection under NIR-II guidance identified metastatic LNs measuring approximately 1 mm (Fig. 7I), which displayed intense fluorescence ex vivo (Fig. 7J). Histopathological analysis confirmed the presence of atypical cells in both tumour and LN specimens. IHC demonstrated variable IFITM1 expression across lesion tissues and metastatic LNs (Fig. 7K–R). These results indicate that IFITM1-IRDye800CW enables sensitive detection and resection of metastatic LNs in orthotopic CRC models under NIR-II fluorescence guidance.

### Imaging performance of IFITM1-IRDye800CW in fresh human CRC specimens

**Fig. 8:**
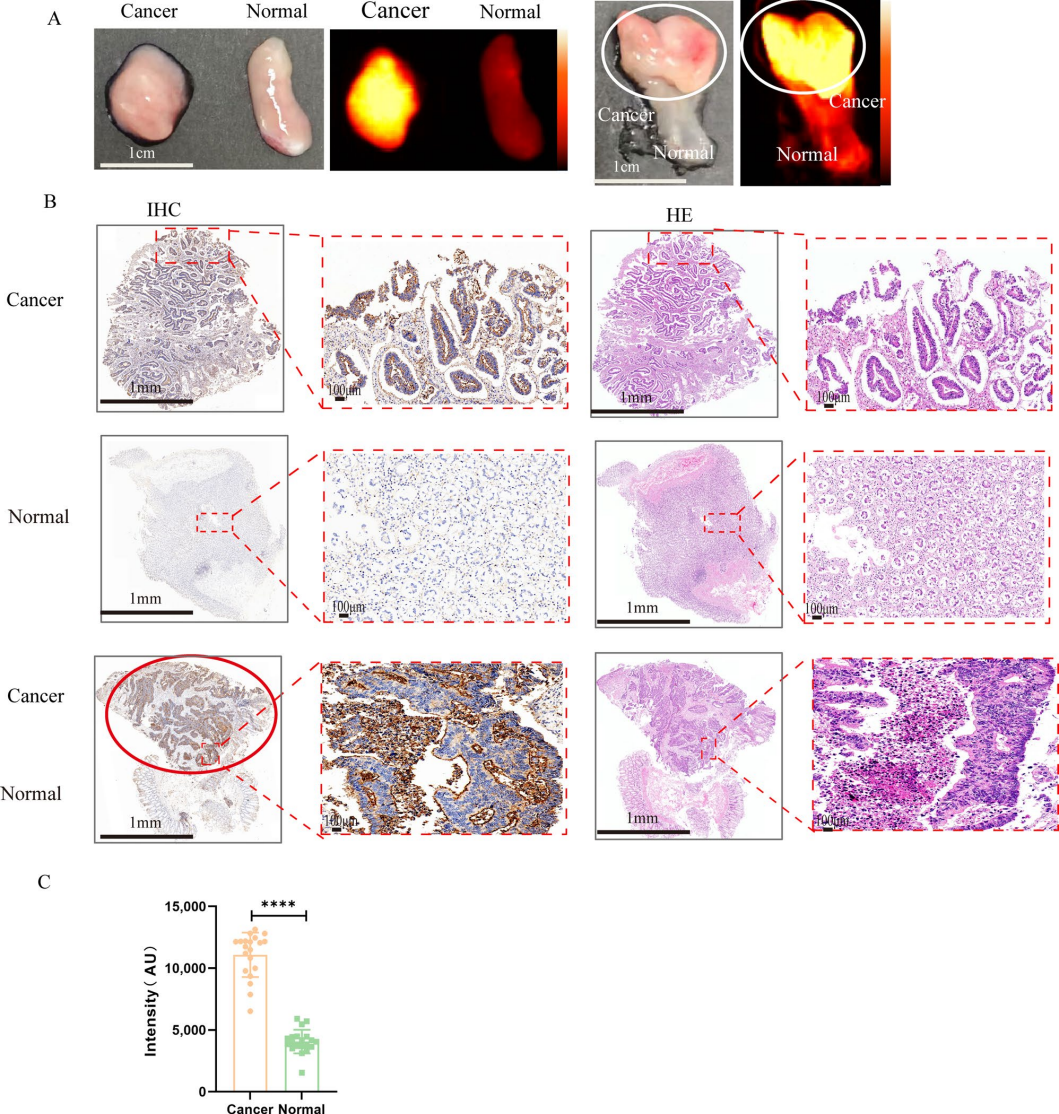
In vitro imaging of fresh human CRC specimens. (**A**) White light and NIR-II fluorescence imaging of surgically resected CRC with normal or adjacent tissues. (**B**) Corresponding histological sections of the same tissues stained with H&E and IHC. (**C**) Statistical analysis of fluorescence intensity between CRC and normal intestinal mucosa. *P* < 0·0001. Scale bars: 1 mm, 100 μm

Twenty pairs of freshly resected CRC specimens were incubated with IFITM1-IRDye800CW under light-protected conditions and imaged using NIR-II fluorescence (Supplement Table 1). Tumour tissues exhibited significantly stronger fluorescence than normal or adjacent para-cancerous tissues (*P* < 0·0001; Fig. 8A, C). High fluorescence signals were observed in 18 of the 20 tumour samples. Histopathological evaluation by H&E staining and IHC confirmed that strong fluorescence corresponded to tumour regions, whereas weak or absent signals aligned with normal tissues (Fig. 8B). IHC further verified high IFITM1 expression in tumour tissues, while normal or adjacent non-cancerous tissues displayed low or absent expression. These findings demonstrate that IFITM1-IRDye800CW provides clear delineation between CRC and adjacent tissues under NIR-II fluorescence, highlighting its potential for intraoperative precision resection and achievement of negative surgical margins.

## Discussion

Advances in fluorescence imaging technology for medical diagnostics have greatly expanded the role of fluorescence-guided procedures in surgery. Most current research focuses on NIR-I imaging, yet its limitations—including shallow tissue penetration (< 1 cm), restricted spatial resolution, high background noise-—impede accurate lesion identification and resection [27]. By contrast, the emerging NIR-II platform, supported by highly sensitive NIR-II cameras, offers improved spatial resolution, imaging depth, and acquisition speed, enabling real-time dynamic visualisation. Its advantages include tissue penetration exceeding 1 cm, high spatial resolution that minimises photon scattering, superior signal-to-noise ratio, low tissue autofluorescence, and enhanced signal-to-background ratio [28–30]. Zhenhua et al. [31] first applied NIR-II imaging to liver cancer resection, demonstrating its superiority over NIR-I in detecting small lesions. NIR-II overcomes the inherent constraints of NIR-I, particularly limited penetration and inadequate visualisation [32–34], and is rapidly progressing from laboratory research to intraoperative application. It is poised to become the next-generation optical platform for precision surgery and early cancer diagnosis.

In this study, we synthesised an NIR-II fluorescent probe, IFITM1-IRDye800CW, targeting CRC, and evaluated its performance in vitro and in vivo. Our findings can be summarised as follows: (1) IFITM1 was highly expressed in CRC tissues but minimally expressed in normal tissues, supporting its suitability as a molecular target. (2) IFITM1-IRDye800CW provided superior NIR-II imaging performance, and improved TBR (2·36 for NIR-II vs. 1·84 for NIR-I). (3) The probe bound specifically to IFITM1-positive CRC cells, with negligible binding to normal cells or IFITM1-low tumours. (4) Following intravenous injection, the probe rapidly accumulated in tumour regions within 6 h. Real-time FGS performed 24 h post-injection demonstrated durable tumour fluorescence for up to 72 h. (5) NIR-II fluorescence enabled accurate identification and resection of metastatic LNs as small as 1 mm. (6) In vitro analysis of fresh CRC specimens confirmed specific binding of the probe to IFITM1-positive tumour samples.

This study incorporated bioinformatics analysis to evaluate IFITM1 expression, revealing that it is highly expressed in colorectal tumours but shows low expression in normal tissues. High IFITM1 expression was further validated in CRC specimens. Growing evidence highlights the critical role of IFITM1 in viral infections and tumour diagnostics. For example, intranasal administration of exogenous soluble IFITM1 (shIFITM1) significantly reduced nasopharyngeal carcinoma infection rates [35]. In addition, rabbit anti-human IFITM1 mAb reagents have been used for gastric cancer tissue detection via immunohistochemistry and have received in vitro diagnostic product (ICD) registration. Other clinical trials targeting IFITM1 are underway [15], The marked differential expression of IFITM1 between tumour and normal tissues supports its potential as a fluorescent targeting agent that provides high-contrast visualisation in the surgical field.

We selected an IFITM1-specific mAb because of its efficient targeting, strong affinity, and high sensitivity, all of which enhanced its ability to localise tumour tissues. IRDye800CW was chosen for its high signal-to-noise ratio, strong spatial resolution, and extended emission tail into the NIR-II spectrum. These properties have produced encouraging results in multiple clinical studies [36–38].

 In vivo imaging experiments demonstrated that IFITM1-800CW NIR-II fluorescence could distinguish subcutaneous tumours with different target expression levels. Tumour TBR values confirmed significant fluorescence differences between tissues with varying IFITM1 expression, underscoring the strong targeting ability of the probe. In vivo and ex vivo organ studies revealed prominent fluorescence in the liver, indicating that IFITM1-IRDye800CW is predominantly metabolised via hepatic pathways. We further established an orthotopic CRC mouse model to replicate the surgical challenge of distinguishing tumour from normal tissue during R0 resection. Tumour fluorescence reached its peak 24 h after probe injection. Under NIR-II imaging, tumour margins were clearly delineated, enabling more precise excision of primary lesions. These findings suggest that the optimal surgical window for guided resection is approximately 24 h post-administration, with the extended fluorescence duration providing flexibility for surgical scheduling. LN metastasis is a key determinant of CRC prognosis. In a cohort of 767 patients higher IFITM1 expression correlated with increased LN metastasis [23, 39]. We therefore hypothesise that IFITM1-800CW not only facilitates accurate localisation and excision of CRC lesions, but also enables sensitive detection of metastatic LNs. Unlike previous models such as the paw-pad system [20], which poorly replicate human LN metastasis, our orthotopic CRC LN metastasis model closely mimics the human in situ tumour microenvironment. NIR-II imaging in this model allowed the detection of metastatic LNs as small as approximately 1 mm, enabling identification of millimetre-scale lesions. Together, these findings support the utility of IFITM1-targeted tracers for imaging both primary and metastatic CRC lesions. - Such targeted fluorescent probes offer broad clinical potential by improving intraoperative decision-making, defining resection boundaries, guiding LN clearances. Ultimately, this approach has the potential to reduce local recurrence and improve patient prognosis [40, 41].

To validate the clinical feasibility of this study, tissue specimens from 20 patients with surgically resected CRC were analysed. Previous studies have shown that incubating fluorescent probes with clinical samples enables tumour identification by NIR-II imaging [22]. Similarly, following incubation of IFITM1-800CW under light-protected conditions, CRC tissues exhibited significantly higher fluorescence intensity than both normal and peritumoural tissues (*P* < 0·0001), with clear demarcation between tumour and adjacent regions. Pathological examination confirmed that strong fluorescence signals corresponded to tumour tissues, whereas weak or absent signals were associated with normal or peritumoural tissues. IHC further validated high IFITM1 expression in tumour regions. These findings demonstrate that IFITM1-targeted tracers can accurately identify CRC, enabling semi-quantitative margin assessment by NIR-II imaging and delineating tumour boundaries. Such molecular fluorescence imaging offers a controllable and standardised approach for clinical application, thereby supporting intraoperative decision-making [41].

This study addresses the clinical challenge of intraoperatively identifying tumours and metastatic LNs in CRC while offering a strategy to reduce the high rate of positive surgical margins. Bevacizumab-800CW, targeting VEGFA, has previously been evaluated in patients with locally advanced cancer undergoing neoadjuvant therapy, where intraoperative NIR fluorescence imaging distinguished tumours from normal tissues and improved detection of tumour-positive circumferential margins (CRMs) [41]. The advent of NIR-II imaging -providing a promising approach to reduce positive margins during CRC surgery. -Residual IFITM1-high clones correlated positively with metastatic lesion counts, with elevated IFITM1expression was observed in metastatic lesions [39]. Moreover, our preliminary studies showed reduced LN metastasis following IFITM1 knockdown, whereas control mice exhibited significant LN spread. Together, these findings highlight IFITM1 as a clinically relevant imaging target, with the specificity of IFITM1 tracers combined with NIR-II technology offering substantial promise for fluorescence-guided CRC surgery.

Beyond higher TBR values, the enhanced performance of NIR-II over NIR-I in biomedical imaging stems from the physical principles governing light interaction within biological tissues. First, according to Rayleigh scattering theory, longer wavelengths exhibit lower scattering coefficients [42]. The significant attenuation of scattering as wavelengths extend from the NIR-I region (~ 800 nm) to the NIR-II region (~ 1500 nm) provides the physical foundation for NIR-II imaging to achieve micrometer-level ultra-high spatial resolution. This enables clearer visualization of subtle tumour boundaries and vascular structures [43]. Second, the NIR-II window exhibits low autofluorescence background, which remains significant in the NIR-I range. In contrast, such autofluorescence is nearly negligible in the NIR-II range, conferring exceptionally high inherent signal-to-noise ratio to NIR-II imaging [44]. With virtually zero background noise, NIR-II imaging demonstrates extreme sensitivity to faint fluorescent signals. In colorectal cancer models with low IFITM1 expression, NIR-I imaging often struggles to detect clear tumour signals due to insufficient signal-to-noise ratio. However, even with low IFITM1 expression levels, NIR-II imaging successfully identified these low-expression lesions despite their weaker signal intensity and less distinct borders compared to high-expression tumour, owing to its extremely low autofluorescence background. This advantage of NIR-II holds promise for reliably detecting tumour with low expression levels, overcoming the bottleneck of weak signals and insufficient contrast in tumour with low target abundance that traditional imaging faces.

This study has several limitations. First, the enrolled patients were not followed longitudinally, precluding assessment of long-term outcomes. Second, although NIR-II imaging data from both mouse models and clinical samples were acquired, no statistical analyses of survival, recurrence, or prognosis were performed in mice. Third, while CRC specimens were analysed, metastatic LNs form patients were too small, and tumour cell distribution within them was uneven, limiting pathological evaluation and preventing clinical validation of LN imaging efficacy, which was instead assessed only in animal models. Fourth, the overall dataset was relatively limited and requires confirmation in larger cohorts. Finally, although laparoscopic surgery is standard practice for CRC, open abdominal imaging was used in this study, as current NIR-II navigation systems primarily support open procedures. The ongoing development of NIR-II fluorescence endoscopes is expected to facilitate translation into minimally invasive surgery.

## Conclusions

In summary, we developed an IFITM1-800CW NIR-II fluorescent probe that enables real-time identification of CRC and metastatic LNs. This approach allowed precise intraoperative delineation of tumour margins and differentiation of tumour from normal tissue, supporting fluorescence-guided R0 resection. IFITM1-targeted NIR-II imaging therefore holds significant potential for reducing positive surgical margins and improving outcomes in patients with CRC.

## Supplementary Information

Below is the link to the electronic supplementary material.

Supplementary Material 1

Supplementary Material 2

Supplementary Material 3

Supplementary Material 4

Supplementary Material 5

Supplementary Material 6

## Data Availability

All data within this paper are available in TCGA database (https://portal.gdc.cancer.gov) and GEPIA database (http://gepia2.cancer-pku.cn/#/index).

## Declarations

### Data sharing statement

The main data supporting the results of this study are available in the paper and its supplementary materials. The raw near-infrared fluorescence images can be obtained after asking the corresponding authors and clarifying the purpose of use.

## Abbreviations

CRC: Colorectal cancer
IFITM1: Interferon-induced transmembrane protein 1
H&E: Haematoxylin and eosin
IHC: Immunohistochemistry
LN: Lymph node
BLI: Bioluminescence imaging
MRI: Magnetic resonance imaging
NIR-I: First near-infrared window
NIR-II: Second near-infrared window
TBR: Tumour-to-background ratio
DAPI: 4′,6-diamidino-2-phenylindole
DiI 1: 1′-dioctadecyl-3,3,3′,3′-tetramethylindocarbocyanine perchlorate
DLS: Dynamic light scattering
ROC: Receiver operating characteristic
TCGA: The Cancer Genome Atlas
GEPIA: Gene Expression Profiling Interactive Analysis
