# Supplementary figures and images for: IFITM1-targeted NIR-II fluorescence imaging enables visualisation of colorectal cancer and metastatic lymph nodes
Source: J Transl Med. 2026 Mar 24;24:618. doi: 10.1186/s12967-026-07938-0 (PMC13134288; doi:10.1186/s12967-026-07938-0)

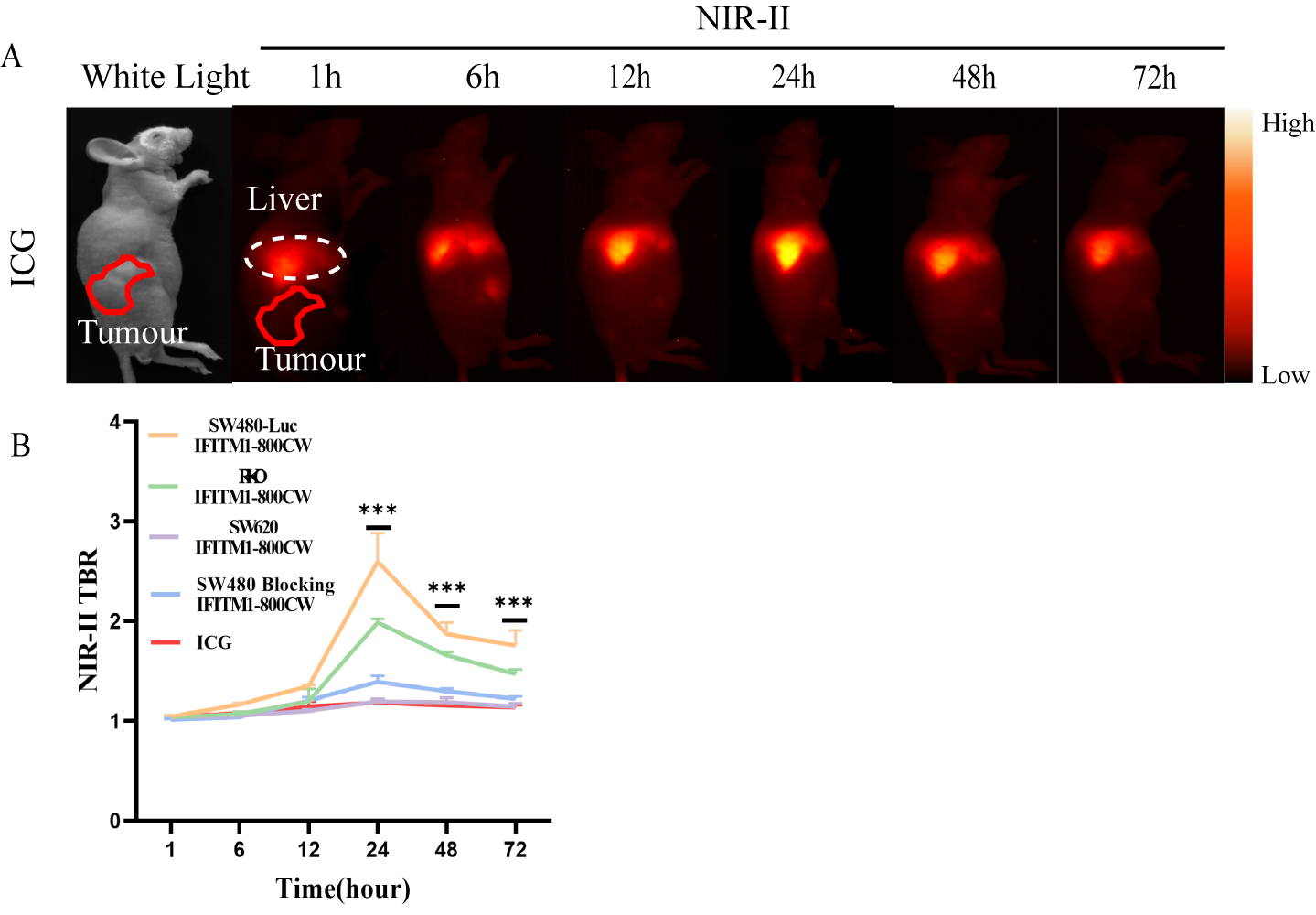

Supplement: Supplementary file 1 — Supplementary Material 1 [file 12967_2026_7938_MOESM1_ESM.tif]

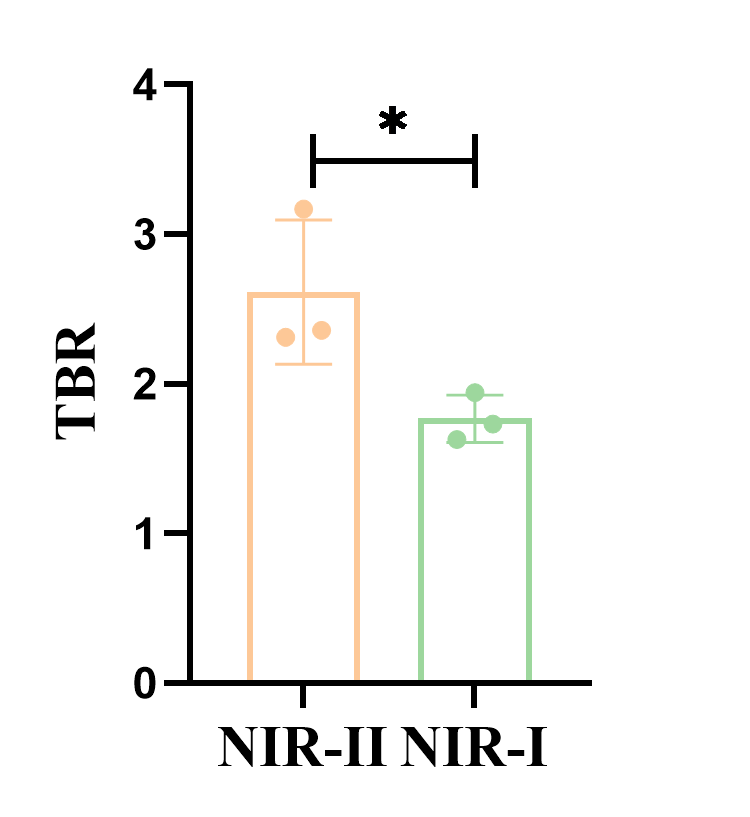

Supplement: Supplementary file 2 — Supplementary Material 2 [file 12967_2026_7938_MOESM2_ESM.tif]
