# Supplementary material for: IFITM1-targeted NIR-II fluorescence imaging enables visualisation of colorectal cancer and metastatic lymph nodes
Source: J Transl Med. 2026 Mar 24;24:618. doi: 10.1186/s12967-026-07938-0 (PMC13134288; doi:10.1186/s12967-026-07938-0)
Supplement: Supplementary file 3 — Supplementary Material 3 [file 12967_2026_7938_MOESM3_ESM.docx]

| Table 1: Patient and tumour characteristics | |
| --- | --- |
| Characteristic |  |
| Age |  |
| ＜60 | 9（45%） |
| ≥60 | 11（55%） |
| Sex |  |
| Men | 16（80%） |
| WoMen | 4（20%） |
| Race |  |
| Asian | 20(100%) |
| Postoperative pathologic staging (AJCC) |  |
| I | 3（15%） |
| II | 6（30%） |
| III | 8（40%） |
| IV | 3（15%） |
| T stage |  |
| 1 | 0 |
| 2 | 5（25%） |
| 3 | 7（35%） |
| 4a | 3（15%） |
| 4b | 5（25%） |
| N stage |  |
| N0 | 9(45%) |
| N1 | 6(30%) |
| N2 | 5(25%) |

AJCC:‌American Joint Committee on Cancer‌

Supplement Table 1 :Patient and tumour characteristics. We collected 20 colorectal cancer tumor specimens.
