# Supplementary material for: IFITM1-targeted NIR-II fluorescence imaging enables visualisation of colorectal cancer and metastatic lymph nodes
Source: J Transl Med. 2026 Mar 24;24:618. doi: 10.1186/s12967-026-07938-0 (PMC13134288; doi:10.1186/s12967-026-07938-0)
Supplement: Supplementary file 4 — Supplementary Material 4 [file 12967_2026_7938_MOESM4_ESM.docx]

**Supplementary material**

**Figure legends**

Figure S1 Evaluation of IFITM1-IRDye800CW specificity and biodistribution in vivo under NIR-II imaging.

1. NIR-II fluorescence imaging of these groups following IFITM1-IRDye800CW injection at 1, 6, 12, 24, 48, and 72 h post-injection. (B) Analysis the trend in TBR changes over 1–72 hours between the SW480-Luc, RKO, SW620，and ICG experimental groups and the SW480-Luc control group, with between-group comparison of TBR.

Figure S2 Quantitative comparison chart of TBR between NIR-II and NIR-I imaging.

Supplement Table 1 :Patient and tumour characteristics. We collected 20 colorectal cancer tumor specimens.
