# Supplementary material for: IFITM1-targeted NIR-II fluorescence imaging enables visualisation of colorectal cancer and metastatic lymph nodes
Source: J Transl Med. 2026 Mar 24;24:618. doi: 10.1186/s12967-026-07938-0 (PMC13134288; doi:10.1186/s12967-026-07938-0)
Supplement: Supplementary file 5 — Supplementary Material 5 [file 12967_2026_7938_MOESM5_ESM.docx]

**Supplementary material of animal studies**

**Housing and husbandry:**

All animals were housed in the SPF room, the location is at the Fujian Cancer Hospital. The feed used for mice was autoclaved and the drinking water was sterilised. Six mice are housed in each box. We check the boxes twice a day on Mondays, Wednesdays and Fridays (early in the morning and late in the evening) and change the mouse litter and clean the cages every three days as needed.

**Animal care and monitoring:**

After inoculation with tumour cells, the tumour growth process is regularly observed every 2-3 days, the tumour size is measured, and the experiment is carried out immediately when the tumour grows to a suitable size. During the course of the experiment, if the weight of the tumour in a mouse exceeds 10% of its body weight, or if the average diameter of the tumour exceeds 20 mm and skin breakdown or ulceration occurs, causing infection or necrosis, the experiment will not be carried out and the animal will be euthanised.
