# Supplementary material for: IFITM1-targeted NIR-II fluorescence imaging enables visualisation of colorectal cancer and metastatic lymph nodes
Source: J Transl Med. 2026 Mar 24;24:618. doi: 10.1186/s12967-026-07938-0 (PMC13134288; doi:10.1186/s12967-026-07938-0)
Supplement: Supplementary file 6 — Supplementary Material 6 [file 12967_2026_7938_MOESM6_ESM.docx]

**Supplementary material of Western blots**

SW62000

SW4800

NCM460

RKO

HT29


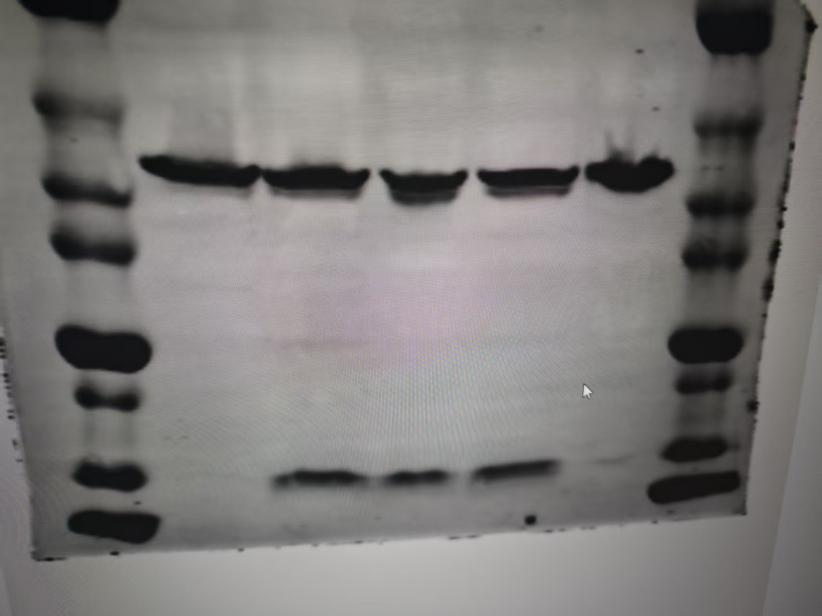


β-actin

IFITM1

40kda

50kda

35kda

25kda

20kda

15kda

10kda

70kda

The original Western blots file of Figure.4A
